# Is checklist an effective tool for teaching research students? A survey-based study

**DOI:** 10.1186/s12909-022-03632-z

**Published:** 2022-07-20

**Authors:** Abdelrahman M Makram, Julia Wang, Gladson Vaghela, Dhir Gala, Nguyen Khoi Quan, Nguyen Tran Minh Duc, Atsuko Imoto, Kazuhiko Moji, Nguyen Tien Huy

**Affiliations:** 1 School of Public Health, Imperial College London, London, UK; 2 Faculty of Medicine, October 6 University, Giza, Egypt; 3 Online Research Club (http://www.onlineresearchclub.org), Nagasaki, Japan; 4 School of Medicine, American University of the Caribbean, Cupecoy, Sint Maarten; 5 GMERS Medical College, Gandhinagar, Gujarat India; 6 College of Health Sciences, VinUniversity, Hanoi, 100000 Vietnam; 7 Faculty of Medicine, University of Medicine and Pharmacy at Ho Chi Minh City, Ho Chi Minh City, Vietnam; 8 School of Tropical Medicine and Global Health, Nagasaki University, 1-12-4 Sakamoto, Nagasaki, 852-8523 Japan; 9 School of Global Humanities and Social Sciences, Nagasaki University, 1-12-4 Sakamoto, Nagasaki, 852-8523 Japan

**Keywords:** Research education, Checklist, Teaching improvement

## Abstract

**Background:**

Students face hardships in determining what are the main points that need more studying in every subject. Checklists are one of the ways that can help students identify the most important pieces of information. Accordingly, in this study, we aimed at examining the impact of using educational checklists on the learning process of postgraduate students at Nagasaki University, Japan.

**Methods:**

Thirty-one Master's students, who finished a “how to write a research protocol” course were recruited by sending them an invitation email that had an attached link to a previously developed and tested questionnaire on the SurveyMonkey® platform. After signing the electronic informed consent, twenty-two participants (response rate = 71%) finished the survey. The data was analyzed using Microsoft Excel and expressed in the form of frequencies and percentages.

**Results:**

More than half of the students declared that they know the checklist will be used in the course that we investigated. Only two students used checklists as a means of studying (9%). Twelve students (55%) confirmed that no other courses or lessons in the School of Tropical Medicine and Global Health (TMGH) use checklists. No students found the usage of checklists not easy or not practical to apply. Many students thought the length of the checklist was suitable and not too short (64%), although three students (14%) found it lengthy. Moreover, most students described the checklist as beyond good (86%) and they would recommend using a checklist for teaching other college students (73%).

**Conclusion:**

Using checklists in education can facilitate the learning process, help in memorization, and deepen the concepts being studied. Further studies are required to examine the impact of checklists in teaching undergraduate students and students from other non-healthcare disciplines.

**Supplementary Information:**

The online version contains supplementary material available at 10.1186/s12909-022-03632-z.

## Key messages

- Educational checklists are tools to help cover all aspects of the taught material.
- Educational checklists help in memorization and deepen the concepts being studied.
- Educational checklists should be simple and to the point.

## Introduction

Checklists provide transparency, organization, and reduction in the risk of human error [1]. They have been successfully implemented in many different processes specifically for these reasons. Surgeons utilize checklists for the health and safety of their parents, pilots for the protection of their planes and passengers, and most of us for simple “to-do” lists [2]. Educational checklists are specifically tailored toward academia, in that they are tools that set out specific criteria, which educators and students may use to gauge skill development or progress and to support the learning process [3]. In that sense, a checklist can be designed for each lecture or lesson to list of the main content the students should focus on.

Because various challenges arise when acquiring knowledge or skills that will help in real-life situations, educational checklists have been used in various settings all over the world for years. One study has shown that having a checklist to assess individual Doctor of Pharmacy (PharmD) students’ performance in a group has proven to be a reliable and valid tool in the setting of small group sessions in a PharmD curriculum [4]. Another study proved that using a checklist represents a promising approach to improving the quality of care for pregnant women with opioid use disorders [5]. Moreover, Wilson and Onwuegbuzie proved that students’ performance and satisfaction improved with the use of structured checklists and scoring guides [2], which may be attributed to actively forgetting non-necessary information, which allows our brains to form and consolidate new memories [6]. In addition, increased stress and emotional distress, which are common in college students, can enhance the forgetfulness pathway [7]. However, the perception of educational checklists by students within the classroom has been rarely investigated, especially in the context of postgraduate studies.

Therefore, this study aimed to evaluate the perception and opinions of students about the usage of a checklist in the development of research protocols. In finding the most tolerable and successful way to teach and assess students in this area, we can improve the completion and success of future research projects as well as improve the way we relay the basics of research to students.

## Methods

### Study setting and participants

The study was based on first-year Master of Tropical Medicine, Master of Public Health, and Master of Science students who had completed a “how to write a research protocol” online course, in March 2021 at Nagasaki University. This is the first time a checklist was used in the teaching of this course. The checklist was distributed electronically during the sessions as each lecture had its own checklist. The students were encouraged to use the checklists in the development of their theses’ protocols. During the examination, the students were also allowed to use the checklist. An example of a checklist used in class is presented in Supplementary Fig. 1.

### Study design

This is a cross-sectional, survey-based study conducted at Nagasaki University, Japan. The reporting of this study followed the Consensus-based Checklist for Reporting of Survey Studies (CROSS) guidelines [8]. The checklist is provided in Supplementary Table 1.

### Questionnaire

The questionnaire was developed by our team, but it was self-administered in English using SurveyMonkey®. Participants were included in this study voluntarily by sending the survey link through an email after the declaration of their results. The questionnaire was available online for 14 days (from June 1^st^ to June 14^th^, 2021)*.* The first page of the questionnaire consisted of the description of the study (including aims, benefits, and the implication of the study) and the informed consent.

The questionnaire is comprised of three sections. The first one included the socio-demographic details (age, gender, highest qualification, and information related to their use of the checklist). The second section included a detailed assessment to evaluate the effect of the checklist on the performance, engagement, and knowledge acquisition by the student. It included questions regarding the structure, benefits, and disadvantages of the checklist. The last section included their final opinion and any recommendations (Additional file 1: Appendix 1).

### Survey conduction

SurveyMonkey® platform was used to administer the survey, within which the informed consent was embedded. All participants were able to decline or withdraw from participation at any time before clicking the submission at the end of the survey. The survey was sent to School of Tropical Medicine and Global Health (TMGH) Master first-year students via email with two reminders sent each week. Any concerns from participants were addressed by NTH.

### Statistical analysis

All the collected data were analyzed using Microsoft Excel. The data were represented as actual frequencies and percentages.

### Ethical considerations

Before sending the participants the enrollment emails, ethical approval was obtained on March 31, 2021, by the Ethical Committee (EC) of the School of Tropical Medicine and Global Health Ethical Committee, Nagasaki University, Japan. The approval number is NU_TMGH_2021_171_1 (Additional file 1: Appendix 2). An electronic informed consent was obtained from each participant before the contribution to the study. They were assured that their participation in this study is completely voluntary and that they can disenroll from the study at any time. If any participants had any questions, they would have contacted the principal investigator (NTH) via email. Moreover, there was no incentive provided to the student for completing the survey.

Confidentiality was preserved by a series of steps conducted that included disabling IP address tracking, not collecting personal information that can lead to the identification of the participants (for example, we collected the age using age groups format, not the exact age), and the data collected was encrypted by SurveyMonkey® before the research team handled the analysis. Finally, the SurveyMonkey® account is only accessible by MDNT and NTH.

## Results

The survey was sent to 31 students with 22 students completing the entire survey: hence, the survey response rate of 71%. The demographics of all the students are presented in Table 1. Out of the 22 students, seven students (32%) were 18–30 years old, and fourteen students (64%) were older than 30 years, while only one student (5%) refused to provide their age. There were ten male students (45%) and twelve female students (55%). Lastly, there were three students (14%) enrolled in the Master of Tropical Medicine, fourteen students (64%) in the Master of Public Health, and five students (23%) in the Master of Science in Global Health and Medicine.

**Table 1:** Baseline characteristics of the participants

	**Response rate**	**Percentage**
**Age**		
18–25 years	1	5%
26–30 years	6	27%
> 30 years	14	64%
Unknown	1	5%
**Total**	**22**	**100%**
**Gender**		
Male	10	45%
Female	12	55%
Unknown	0	0%
**Total**	**22**	**100%**
**Degree**		
PhD	0	0%
Master	16	73%
College/University	6	27%
Unknown	0	0%
**Total**	**22**	**100%**
**Course**		
PhD	0	0%
MTM	3	14%
MPH	14	64%
MSc	5	23%
Unknown	0	0%
**Total**	**22**	**100%**

*Abbreviations*: *Ph.D.* (Doctor of Philosophy degree), *MTM* (Master of Tropical Medicine), *MPH* (Master of Public Health), *MSc* (Master of Science in Global Health and Medicine)

The results of the survey are included in Table 2. Twelve participants (55%) declared that they knew that the course evaluation would depend on using a checklist that would be made available to everyone throughout the course, three did not know that (14%), six students (27%) stated that they could not recall, and one student (5%) did not respond to the question. Among the students, two (9%) usually used a checklist during their studies before attending TMGH, eight (36%) used a checklist intermittently, five (23%) rarely utilized a checklist, and seven (32%) never used a checklist, and no student reported always using a checklist before attending. Fifteen students (68%) found the checklist practical and easy to apply, while seven students (32%) noted they were not sure, and none of the students declared the checklist was impractical or not easy to apply. The length of the checklist was found to be suitable for its contents by fourteen students (64%), three (14%) found that it was too long, five (23%) stated that they were unsure, and no student thought it to be too short. Gaining a better understanding of the lesson with the aid of the checklist was reported by sixteen students (73%), and thirteen (59%) proclaimed there were no disadvantages to the checklist for their learning. The quality of teaching while using a checklist was found to be perfect by 2 students (9%), good by seventeen (77%), not good or bad by three (14%), and none of the students recorded that the quality was bad or awful. Utilizing a checklist for instructing college students was recommended by sixteen of the students (73%), and six (27%) said that maybe they would recommend this teaching style.

**Table 2:** Participants’ responses to the survey

Assessing the Checklist	Response rate	Percentage
**Were you informed at the beginning of the course that a checklist would be used to evaluate the progress of the course throughout its duration?**		
Yes	12	55%
No	3	14%
Can’t Recall	6	27%
Unknown	1	5%
**Total**	**22**	**100%**
**Have other professors of TMGH used checklists in your courses or lessons?**		
Never	7	32%
Rarely	5	23%
Sometimes	7	32%
Usually	2	9%
Always	1	5%
**Total**	**22**	**100%**
**Have you used or do you use checklists in other studies before attending TMGH?**		
Never	7	32%
Rarely	5	23%
Sometimes	8	36%
Usually	2	9%
Always	0	0%
**Total**	**22**	**100%**
**Was the checklist practical and/or easy to apply?**		
Yes	15	68%
No	0	0%
Not Sure	7	32%
**Total**	**22**	**100%**
**Was the length of the checklist suitable enough for the contents in it?**		
Yes, it was good	14	64%
No, it was too long	3	14%
No, it was too short	0	0%
Not sure	5	23%
**Total**	**22**	**100%**
**Having a checklist in class has helped me… (select all that apply)**		
Concentrate better in class	7	31.82%
Understand better the lesson and knowledge	16	72.73%
Better for the class	11	50.00%
Study better for the final exam	10	45.45%
Keep focused and prevent unnecessary detours in the course information	10	45.45%
Write the protocol more systematically and organizationally	13	59.09%
Finish writing the protocol in less time	3	13.64%
Communicate better with my colleagues	8	36.36%
Teach another student how to write a protocol easily	3	13.64%
Save my time to remember all items and sections	8	36.36%
The checklist did not provide any significant advantages	1	4.55%
**Please select all the disadvantages you found in being taught using a checklist**		
The checklist has eliminated my constant need to ask for the instructor’s help	3	13.64%
The checklist did not cover all the points required to complete a protocol	3	13.64%
The checklist was so lengthy that whenever I try to use it, I get confused	5	22.73%
Using the checklist lengthened the time needed for writing a protocol	2	9.09%
I found no disadvantages to the checklist	13	59.09%
**How would you describe the overall quality of the checklist teaching?**		
Perfect	2	9%
Good	17	77%
Not good not bad	3	14%
Bad	0	0%
Awful	0	0%
**Total**	**22**	**100%**
**Would you recommend using a checklist for teaching college students?**		
Yes	16	73%
No	0	0%
Maybe	6	27%
**Total**	**22**	**100%**

*Abbreviations*: *TMGH* (School of Tropical Medicine and Global Health, Nagasaki University, Japan)

Finally, only twenty students responded to the final opinion questions. In response to whether the checklist had all items listed, two students were “not sure”. One student stated that the “institution of the researcher [for which] the protocol [is to be submitted]” was missing, and another one wanted to include an item about “the issue that [the] student needs to [research] about”, which we anticipated to mean the knowledge gap. The rest thought that the checklist “covered all points”. As for their final opinions, one student stated that the “checklist is really helpful, but [they] think it will be better if there is certain explanation or instruction prior to [using] it”. Another one stated that “it is important to review the checklist at regular intervals”. A third one said agreed that a “checklist is a summary of a research so it’s important especially when making research plans”. A fourth specified that “the checklist is very practical to apply [in] both theoretical and field knowledge”. A fifth thought that a checklist is “the best way of teaching with this struggling time”, they probably meant during the COVID-19 pandemic, which coincided with the usage of the checklist. And a final one approved of the usage of a checklist as “a good tool”.

## Discussion

Our cross-sectional, quantitative, survey-based study was conducted at Nagasaki University, Japan in 2021. With this study, we aimed at investigating the students’ perception of using checklists in class and the necessity of having a checklist to aid learning. It was not surprising to discover that most students do not use checklists in their courses so often, as only two of them (9%) usually used them. Twelve students confirmed that no other courses or lessons in TMGH use checklists. However, no students found the usage of checklists not easy or not practical to apply, and many of them thought that the length of the checklist was suitable and not too short, although three students (14%) found it quite lengthy.

Both teaching and learning medical education content are challenging. A common standard is needed within the medical education system to aid in the improvement of both processes [9, 10]. Checklists are widely used and have shown to be effective in various fields [4, 5]. It is not only a valuable tool as perceived by medical students [11], but it also helps practitioners in dealing with complex situations and crises [12]. Researchers have also found that checklists can reduce morbidity and mortality in surgery [13] and increase the adherence of anesthesiologists to guidelines and non-technical skills in simulations of emergencies [14]. Checklists also allow for comprehensive, well-organized, and efficient assessment of the steps conducted in a certain process, which can foster process optimization and creativity [5, 11, 14]. Lastly, using a checklist can avoid missing critical steps in practice [14].

Health education relies largely upon passing knowledge from the instructor to the student. To properly train future healthcare workers and researchers, we must broaden our understanding of how to teach, notably, by including intentional instructional design in the way curriculum is carried out, as a component of clinical education [15]. Undoubtedly, checklists are among the best methods for instructional design studies and their protocols. As for demonstrating the necessity of a checklist, our study has shown four major fields students found improvement in as a direct result of its implementation. These areas were: understanding the lesson and curriculum knowledge, writing the protocol systematically and organizationally, studying for the final exam, and keeping focused and preventing unnecessary detours in the course information. Our results were like a study of De La Garza et al., which revealed that checklists also help increase students’ knowledge acquisition with E-learning [16]. More than one-third of our participants responded that the checklist helped them to not only save time remembering details but also facilitate communication between colleagues (*n* = 8, accounted for 36%). A total of seven students (32%) were able to concentrate better in class. Only one student found the checklist did not provide any significant advantages, which accounted for 5%. No students found the checklist was bad or awful. Most of them described the checklist as beyond good (*n* = 19; 86%) and they would recommend using a checklist for teaching other college students (*n* = 16; 73%).

Some limitations of checklists were also investigated. One common disadvantage was that the checklist was so lengthy that it confused students (*n* = 5; 23%). To a lesser extent, three students (14%) found the checklist was not thorough because of some missing information, and 3 others complained that the checklist has eliminated their need to ask for instructors’ help. In an educational environment, communication between students and teachers/instructors is a crucial part of learning but is often limited. Checklists can further degrade this relationship. We suggest that students use checklists as an organizational tool and a reference source and not for sole dependence on information to resolve this issue. That is because more than the half of students (*n* = 13, 59%) were satisfied with the checklist and didn’t find any disadvantages. We were also not objectively able to assess the efficacy of checklists on the educational progress of students as including the grades in the survey may have jeopardized the confidentiality and further decreased the participation rate. We were also not able to assess the usage of checklists qualitatively (through interviews) because of some ethical concerns raised by the Ethical Committee at the School of Tropical Medicine and Hygiene, Nagasaki University. In addition, we have not examined the effect of a checklist on the educational attainment of undergraduate students. Conducting further studies with a bigger sample that includes other medical or research courses is essential to explore the true impact of using educational checklists and the true perception and opinion of students regarding them.

Despite the limitations, our pilot study revealed students have a positive perception of using checklists in class. In the range of our knowledge, our study was the first study in the field of using checklists in education, especially within an educational research setting. The results also suggested that using checklists can ameliorate overall education quality, help to keep students focused and improve their knowledge acquisition, and reduce time-consuming tasks, all the while facilitating communication. It is worth mentioning that a practitioner’s education should be multifaceted and not dependent upon one strategy, such as checklists alone. Furthermore, checklists should be used wisely and alongside other education strategies. For example, a checklist could be used as a supplement for the *Rigorous* section of the “10R’s of Clinician Education”, to attain maximum effect [15]. Although there might be some generalizability concerns about this study, we believe that this work will pave the way for further studies with better designs to investigate the pros and cons of using checklists within a classroom setting. This should include more qualitatively collected data to better reflect the opinion and comments of the students about the checklist itself and the general usage of checklists in the educational system. With the information provided in this paper and its supplementary material, the authors believe that the results of this study can be easily replicated in other institutions as we are aware that checklists are used in many education entities around the world. In laying this foundation, we hope to ultimately contribute to the improvement in the quality of the medical education system.

## Conclusion

In this study, we aimed at exploring the benefits of using a checklist in research education. This was done by surveying 22 Master's students who attended a “how to write a research protocol” course at Nagasaki University, Japan. All students agreed that the usage of the checklist was not difficult or impractical. And most of them described the checklist as beyond good and recommended its use for teaching other college subjects. Finally, we see that using checklists in education can facilitate the learning process, help in memorization, and deepen the concepts being studied. Further studies are required to examine the impact of checklists in teaching undergraduate students and students from other non-healthcare disciplines.

## Supplementary Information

**Additional file 1:** **Supplementary Table 1.** Checklist for Reporting of Survey Studies (CROSS). **Supplementary Figure 1.** The checklist used for the lecture of “research ethics”. **Appendix 1.** The questionnaire with informed consent was sent to the participants. **Appendix 2.** Ethical committee approval from the School of Tropical Medicine and Global Health Ethical Committee, Nagasaki University, Japan.

## Data Availability

The datasets generated and/or analyzed during the current study are not publicly available because the sample size is not large enough to guarantee the full confidentiality of the participants. Furthermore, we have not obtained consent from the participants to share the raw material. However, the data can be provided by the corresponding author (Nguyen Tien Huy; tienhuy@nagasaki-u.ac.jp) upon reasonable request.

## Abbreviations

TMGH: School of Tropical Medicine and Global Health
PharmD: Doctor of Pharmacy
EC: Ethical Committee
CROSS: Consensus-based Checklist for Reporting of Survey Studies
