# Supplementary material for: Is checklist an effective tool for teaching research students? A survey-based study
Source: BMC Med Educ. 2022 Jul 20;22:561. doi: 10.1186/s12909-022-03632-z (PMC9297669; doi:10.1186/s12909-022-03632-z)

**Is Checklist an Effective Tool for Teaching Research Students? A Survey-based Study**

**Supplementary material**

**Supplementary Table 1.** Checklist for Reporting of Survey Studies (CROSS).

**Supplementary Figure 1.** The checklist used for the lecture of “research ethics”.

**Appendix 1.** The questionnaire with informed consent was sent to the participants.

**Appendix 2.** Ethical committee approval from the School of Tropical Medicine and Global Health Ethical Committee, Nagasaki University, Japan.

**Supplementary Table 1.** Checklist for Reporting of Survey Studies (CROSS).

| **Section/topic** | **Item** | **Description** | **Reported on page #** |
| --- | --- | --- | --- |
| **Title and abstract** | | |  |
| **Title and abstract** | 1a | State the word “survey” along with a commonly used term in title or abstract to introduce the study’s design. | 1 |
|  | 1b | Provide an informative summary in the abstract, covering background, objectives, methods, findings/results, interpretation/discussion, and conclusions. | 3 |
| **Introduction** | | |  |
| **Background** | 2 | Provide a background about the rationale of study, what has been previously done, and why this survey is needed. | 4 |
| **Purpose/aim** | 3 | Identify specific purposes, aims, goals, or objectives of the study. | 5 |
| **Methods** | | |  |
| **Study design** | 4 | Specify the study design in the methods section with a commonly used term (e.g., cross-sectional or longitudinal). | 6 |
|  | 5a | Describe the questionnaire (e.g., number of sections, number of questions, number and names of instruments used). | 6 |
| **Data collection methods** | 5b | Describe all questionnaire instruments that were used in the survey to measure particular concepts. Report target population, reported validity and reliability information, scoring/classification procedure, and reference links (if any). | 6 |
|  | 5c | Provide information on pretesting of the questionnaire, if performed (in the article or in an online supplement). Report the method of pretesting, number of times questionnaire was pre-tested, number and demographics of participants used for pretesting, and the level of similarity of demographics between pre-testing participants and sample population. | 6 |
|  | 5d | Questionnaire, if possible, should be fully provided (in the article, or as appendices or as an online supplement). | Supplementary Table 1 |
| **Sample characteristics** | 6a | Describe the study population (i.e., background, locations, eligibility criteria for participant inclusion in survey, exclusion criteria). | 6 |
|  | 6b | Describe the sampling techniques used (e.g., single stage or multistage sampling, simple random sampling, stratified sampling, cluster sampling, convenience sampling). Specify the locations of sample participants whenever clustered sampling was applied. | 6 |
|  | 6c | Provide information on sample size, along with details of sample size calculation. | 6 |
|  | 6d | Describe how representative the sample is of the study population (or target population if possible), particularly for population-based surveys. | 6 |
| **Survey**  **administration** | 7a | Provide information on modes of questionnaire administration, including the type and number of contacts, the location where the survey was conducted (e.g., outpatient room or by use of online tools, such as SurveyMonkey). | 6 |
|  | 7b | Provide information of survey’s time frame, such as periods of recruitment, exposure, and follow-up days. | 6 |
|  | 7c | Provide information on the entry process:  –>For non-web-based surveys, provide approaches to minimize human error in data entry.  –>For web-based surveys, provide approaches to prevent “multiple participation” of participants. | 6 |
| **Study preparation** | 8 | Describe any preparation process before conducting the survey (e.g., interviewers’ training process, advertising the survey). | 6 |
| **Ethical considerations** | 9a | Provide information on ethical approval for the survey if obtained, including informed consent, institutional review board [IRB] approval, Helsinki declaration, and good clinical practice [GCP] declaration (as appropriate). | 7 |
|  | 9b | Provide information about survey anonymity and confidentiality and describe what mechanisms were used to protect unauthorized access. | 7 |
| **Statistical**  **analysis** | 10a | Describe statistical methods and analytical approach. Report the statistical software that was used for data analysis. | 7 |
|  | 10b | Report any modification of variables used in the analysis, along with reference (if available). | 6 |
|  | 10c | Report details about how missing data was handled. Include rate of missing items, missing data mechanism (i.e., missing completely at random [MCAR], missing at random [MAR] or missing not at random [MNAR]) and methods used to deal with missing data (e.g., multiple imputation). | NA |
|  | 10d | State how non-response error was addressed. | NA |
|  | 10e | For longitudinal surveys, state how loss to follow-up was addressed. | NA |
|  | 10f | Indicate whether any methods such as weighting of items or propensity scores have been used to adjust for non-representativeness of the sample. | NA |
|  | 10g | Describe any sensitivity analysis conducted. | NA |
| **Results** | | |  |
| **Respondent characteristics** | 11a | Report numbers of individuals at each stage of the study. Consider using a flow diagram, if possible. | 8 |
|  | 11b | Provide reasons for non-participation at each stage, if possible. | 8 |
|  | 11c | Report response rate, present the definition of response rate or the formula used to calculate response rate. | 8 |
|  | 11d | Provide information to define how unique visitors are determined. Report number of unique visitors along with relevant proportions (e.g., view proportion, participation proportion, completion proportion). | 8 |
| **Descriptive**  **results** | 12 | Provide characteristics of study participants, as well as information on potential confounders and assessed outcomes. | 8 |
| **Main findings** | 13a | Give unadjusted estimates and, if applicable, confounder-adjusted estimates along with 95% confidence intervals and p-values. | NA |
|  | 13b | For multivariable analysis, provide information on the model building process, model fit statistics, and model assumptions (as appropriate). | NA |
|  | 13c | Provide details about any sensitivity analysis performed. If there are considerable amount of missing data, report sensitivity analyses comparing the results of complete cases with that of the imputed dataset (if possible). | NA |
| **Discussion** | | |  |
| **Limitations** | 14 | Discuss the limitations of the study, considering sources of potential biases and imprecisions, such as non-representativeness of sample, study design, important uncontrolled confounders. | 10 |
| **Interpretations** | 15 | Give a cautious overall interpretation of results, based on potential biases and imprecisions and suggest areas for future research. | 10 |
| **Generalizability** | 16 | Discuss the external validity of the results. | 10 |
| **Other sections** | | |  |
| **Role of funding source** | 17 | State whether any funding organization has had any roles in the survey’s design, implementation, and analysis. | 2 |
| **Conflict of interest** | 18 | Declare any potential conflict of interest. | 2 |
| **Acknowledgements** | 19 | Provide names of organizations/persons that are acknowledged along with their contribution to the research. | 3 |

**Supplementary Figure 1.** The checklist used for the lecture of “research ethics”.


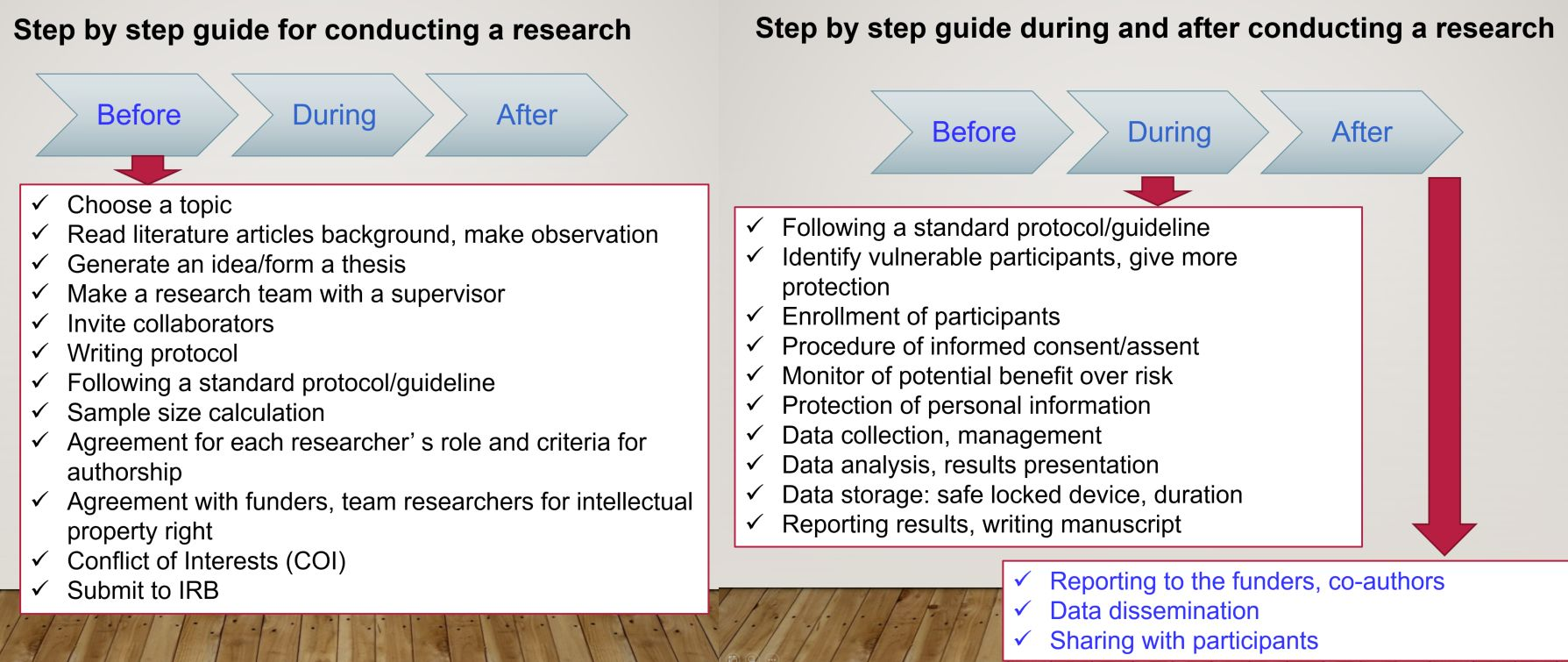


**Appendix 1.** The questionnaire with informed consent was sent to the participants.

**Is Checklist a Good Tool for Teaching Students? A Survey-based Study.**

**Introduction**

We are a group of researchers from the School of Tropical Medicine and Global Health, Nagasaki University (TMGH) who are conducting this study to assess the effectiveness of using a checklist as an educational tool to assess the progress of completion of the course “How to write the protocol” and ethics course, to increase the effectiveness and success of the students and the course. If you enrolled in these courses, you are invited to participate in this survey.

The survey comprises 15 questions, and it may take approximately 5 minutes. You are free to decline or withdraw from participation at any time.

The responses collected from this survey are confidential and will not be revealed under any conditions. Besides, the survey will be completely anonymous. After the study is completed, the data utilized will be encrypted and stored in a password-protected Cloud Drive for at least ten years. There is only a minimal risk such as low stress while reading the survey. Participating in this study may not benefit you directly, but the results may improve future teaching of courses at TMGH that may return significant benefits for the participants.

Please participate in the survey by following the URL below.

For any further queries, you can contact our team by emailing Nguyen Tien Huy at [tienhuy@nagasaki-u.ac.jp](mailto:tienhuy@nagasaki-u.ac.jp)

IMPORTANT NOTE: By completing and submitting this survey, you are indicating your consent to participate in the survey. There is no cost or reimbursement for participating in the study.

**Part I: Personal Information**

1. Please indicate your age.
2. 18 – 25 years old
3. 26 – 30 years old
4. > 30 years old
5. Please provide your gender.
6. Male
7. Female
8. Prefer not to say
9. What degree do you currently hold?
10. PhD degree
11. Master degree
12. College/university/bachelor degree
13. Prefer not to say
14. Which course are you learning?

a. PhD course

b. MTM

c. MPH

d. Master of Science

e. Prefer not to say

**Part II: Assessing the Checklist**

1. Were you informed at the beginning of the course that a checklist would be used to evaluate the progress of the course throughout its duration?
2. Yes
3. No
4. Can’t recall
5. Have other professors of TMGH used checklists in your courses or lessons?
6. Never
7. Rarely
8. Sometimes
9. Usually
10. Always
11. Have you used or do you use checklists in other studies before attending TMGH?
12. Never
13. Rarely
14. Sometimes
15. Usually
16. Always
17. Was the checklist practical and/or easy to apply?
18. Yes
19. No
20. Not sure
21. Was the length of the checklist suitable enough for the contents in it?
22. Yes, it is good.
23. No, it was too long.
24. No, it was too short.
25. Not sure
26. Having a checklist in class has helped me … (please select all that apply)
27. … concentrate better in class.
28. … understand better the lesson and knowledge
29. … prepare better for the class.
30. … study better for the final exam.
31. … keep focused and prevented unnecessary detours in the course information.
32. … write the protocol more systematically and organizationally.
33. … finish writing the protocol in less time.
34. … communicate better with my colleagues.
35. … teach another student how to write a protocol easily.
36. … save my time to remember all items and sections
37. The checklist did not provide any significant advantages.
38. Please select all the disadvantages you found in being taught using a checklist.
39. The checklist has eliminated my constant need to ask for the instructor’s help.
40. The checklist did not cover all the points required to complete a protocol.
41. The checklist was so lengthy that whenever I try to use it, I get confused.
42. Using the checklist lengthened the time needed for writing a protocol.
43. I found no disadvantages to the checklist.

**Part III: Final Opinion**

1. How would you describe the overall quality of the checklist teaching?
2. Perfect
3. Good
4. Not good and not bad
5. Bad
6. Awful
7. Would you recommend using a checklist for teaching college students?
8. Yes
9. No
10. Maybe
11. Do you think there was an important missing point in the checklist?

…

1. Do you have any other comments, suggestions, or recommendations?

…

**Appendix 1.** Ethical committee approval from the School of Tropical Medicine and Global Health Ethical Committee, Nagasaki University, Japan.


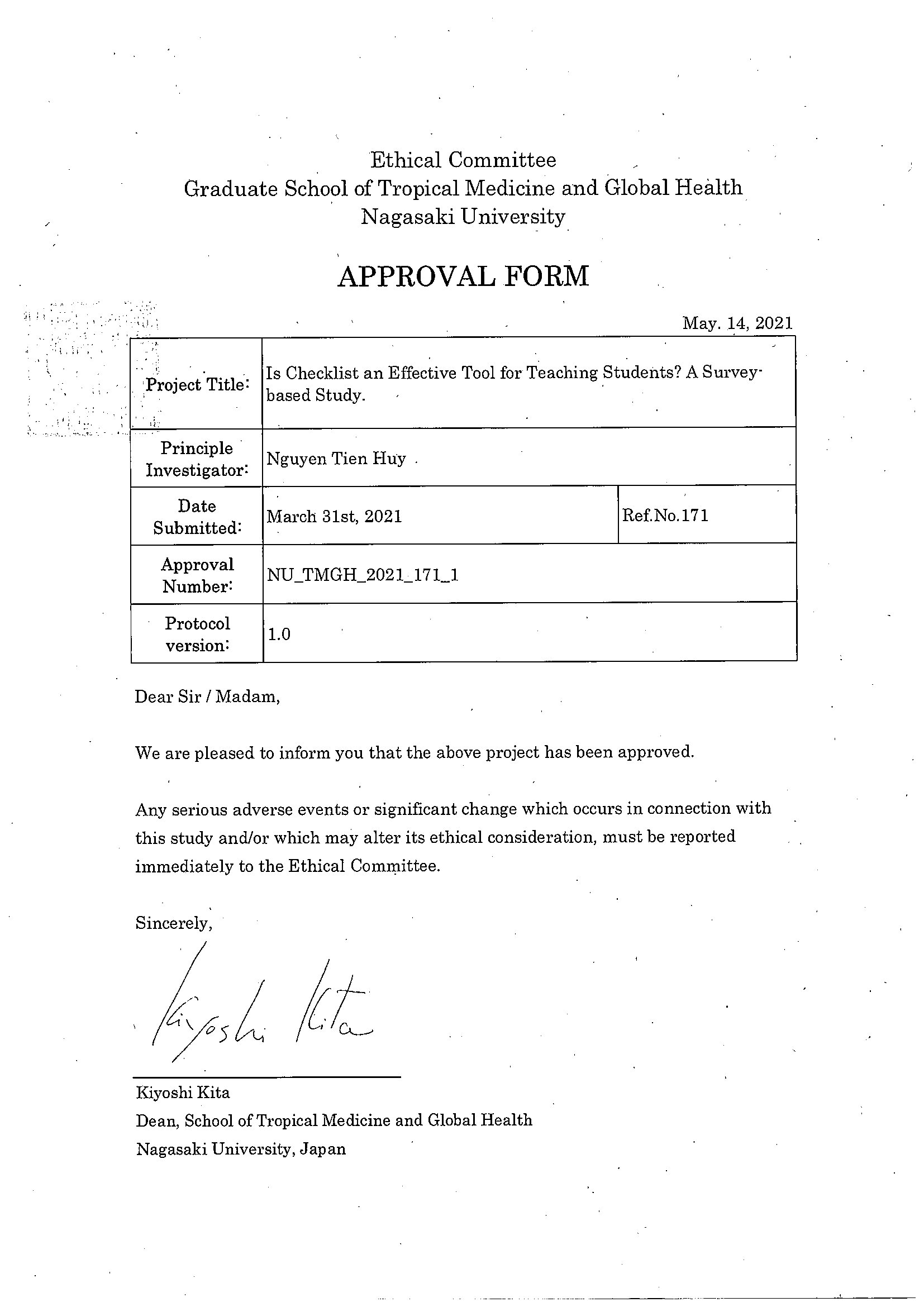

Supplement: Supplementary file 1 — Additional file 1: Supplementary Table 1. Checklist for Reporting of Survey Studies (CROSS). Supplementary Figure 1. The checklist used for the lecture of “research ethics”. Appendix 1. The questionnaire with informed consent was sent to the participants. Appendix 2. Ethical committee approval from the School of Tropical Medicine and Global Health Ethical Committee, Nagasaki University, Japan. [file 12909_2022_3632_MOESM1_ESM.docx]
